# Supplementary material for: Personal and psychosocial factors of burnout: A survey within the French neurosurgical community
Source: PLoS One. 2020 May 29;15(5):e0233137. doi: 10.1371/journal.pone.0233137 (PMC7259549; doi:10.1371/journal.pone.0233137)
Supplement: S1 Appendix — (PDF) [file pone.0233137.s001.pdf]

Health at Work of the French Neurosurgical Community

Dear colleagues,

the profession of doctor is a physically and emotionally demanding activity. Its goal is to preserve and improve the health of patients.
Seniors and interns of neurosurgery, you are engaged in a specialty where the concept of risk is inseparable from the pressure it implies.
The purpose of this questionnaire is to assess the mental health of the neurosurgical community and its determinants, using validated academic tools. The objective is to offer practical and preventive solutions to the difficulties encountered. This approach is part of the report of November 5, 2018 on the recertification of doctors which states that the doctor takes care of his health and quality of life.

This questionnaire is anonymous and the data collected is secure and used for the sole purposes of the study. It was declared to the CNIL (n ° 2211947 v 0) and approved by the ethics committee of the college of neurosurgery on 03/14/2019 (ref. IRB00011687). The result of this study will lead to an original publication.

Thanking you for your collaboration in this survey (less than 15 min) and remaining at your disposal for any further information,

Clément Baumgarten - CHU Grenoble - Resident
Emmanuel Gay - CHU Grenoble - President of the SFNC - PU-PH
Pierre-Hugues Roche - Marseille University Hospital - President of the College of Neurosurgery - PU-PH
Estelle Michinov - Rennes 2 University - Professor of psychology
Géraldine Rouxel - Rennes 2 University - Psychology
*Obligatory

1. About everyday work: *

Only one answer possible per line.

	Disagree	Agree, and I'm not at all disturbed	Agree, and I'm a little confused	Agree, and I'm disturbed	Agree, and I'm very upset
1. I am constantly pressed for time due to a heavy workload	◯	◯	◯	◯	◯
2. I am frequently interrupted and disturbed in my workmpu (e) and disturbed (e) in my work	◯	◯	◯	◯	◯
3. I have a lot of responsibilities at work	◯	◯	◯	◯	◯
4. I am often forced to work overtime	◯	◯	◯	◯	◯
5. My job requires physical effort	◯	◯	◯	◯	◯
6. In recent years my work has become more and more demanding	◯	◯	◯	◯	◯
7. I am treated unfairly in my work	◯	◯	◯	◯	◯
8. Given all my efforts, I receive the respect and esteem I deserve at my work	◯	◯	◯	◯	◯
9. I am living or expecting to experience an undesirable change in my work situation	◯	◯	◯	◯	◯
10. My promotion prospects are weak	◯	◯	◯	◯	◯

11.My job security is at risk					
12. At work, I often get pressed for time					
13. I start thinking about problems at work as soon as I get up in the morning					
14. When I get home, I can easily relax and forget everything about my work					
15. My loved ones say that I sacrifice myself too much for my work					
16. Work is still on my mind when I go to bed					
17. When I put off something that I should do the same day, I have trouble sleeping at night					

2. About everyday work: *

Only one answer possible per line.

	Agree	Disagree, and I am not at all disturbed	Disagree, and I am a little disturbed	Disagree, and I am disturbed	Disagree, and I am very upset
1. I receive the respect I deserve from my superiors					
2. I receive the respect I deserve from my colleagues					
3. At work I have satisfactory support in difficult situations					
4. My job security is at risk					
5. My current professional position corresponds well to my training					
6.Seeing all my efforts, I receive the respect and esteem I deserve for my work					
7. Considering all my efforts, my promotion prospects are satisfactory					

3. About everyday work: specify how often you feel the description of the following proposals. *

Only one answer possible per line.

	Never	Almost never	Sometimes	Regularly	Often	Very often	Always
1. I would still like to do this job, even if I was paid lesse ce travail, even if I was paid less							
2. When I work, I don't think of anything else							
3. My job gives me pleasant feelings							
4. I also want to work in my spare time							
5. I get carried away by my work							
6. I do my job with great pleasure							
7. I work because I like it							
8. When I work, I forget everything							

else around me							
9. I feel happy during my work							
10. When I work on something, I do it for myself							
11. I am totally immersed in my work							
12. I feel happy when I am at work							
13. I get my motivation from the work itself and not from the rewards I get							

4. About everyday work: specify how often you feel the description of the following proposals. *

Only one answer possible per line.

	Never	Sometimes	Often	Always
1. I feel like I'm in a hurry and running against the clock				
2. I stay at work even after my colleagues have left.				
3. It is important for me to work hard even when I don't like what I am doing				
4. I am always busy and I work on many projects at the same time.				
5. It seems to me that I have an internal compulsion that pushes me to work hard, a feeling that it is something that I must do whether I like it or not.				
6. I spend more time working than spending time with friends, having hobbies or playing hobbies.				
7. I feel compelled to work for the same time as what I am doing is not pleasant.				
8. I find myself doing two or three things simultaneously, such as eating my midday meal and writing a note while I am on the phone				
9. I feel guilty when I have time off from work				
10. It's difficult to relax when I'm not at work				

5. About family - work relationships *

Only one answer possible per line.

	Strongly disagree	Disagree	Somewhat disagree	Undecided	Somewhat agree	Agree	Strongly agree
1. My family life encroaches on my professional activities							
2. My family life intrudes on my professional life, preventing me from arriving at work on time, from performing my daily tasks and from working overtime							
3. My professional life intrudes on my private life.							
4. My job prevents me from doing everything I wish I could do at home.							
5. My partners and work colleagues do not appreciate that I am so often concerned about my family life							
6. There is a conflict between my							

work and my family responsibilities							
7. Sometimes I need to take time off work to be able to take on my family responsibilities							
8. My job prevents me from having a harmonious family life							
9. I can't manage to complete certain tasks that my job involves because of the demands of my family / my spouse							
10. I often have to give up important moments in family life because of my work							

6. I consider myself a person who ... *

Only one answer possible per line.

	strongly disagree	disagree a little	neither approve or disapprove	approve a little	strongly approve
1. I see myself as someone who is talkative.					
2. I see myself as someone who tends to criticize others.					
3. I see myself as someone who works conscientiously.					
4. I see myself as someone who is depressed, cockroach.					
5. I see myself as someone who is creative, full of original ideas.					
6. I see myself as someone who is reserved.					
7. I see myself as someone who is helpful and not selfish with others.					
8. I see myself as someone who can sometimes be careless.					
9. I see myself as someone who is "relaxed", relaxed, copes with stress well.					
10. I see myself as someone who is interested in many subjects.					
11. I see myself as someone who is full of energy.					

12. I see myself as someone who easily starts arguing with others.

13. I see myself as someone who is reliable in his work.

14. I see myself as someone who can be anxious.

15. I see myself as someone who is ingenious.

16. I see myself as someone who communicates a lot of enthusiasm.

17. I see myself as someone who is forgiving by nature.

18. I see myself as someone who tends to be disorganized.

19. I see myself as someone who torments himself a lot.

20. I see myself as someone who has a great imagination.

21. I see myself as someone who tends to be silent.

22. I see myself as someone who generally trusts others.

23. I see myself as someone who tends to be lazy.

24. I see myself as a

temperate person, not easily upset.

25. I see myself as someone who is inventive.

26. I see myself as someone who has a strong personality, speaks with confidence.

27. I see myself as someone who is sometimes dismissive, contemptuous.

28. I see myself as someone who perseveres until his task is finished.

29. I see myself as someone who can be moody, moody.

30. I see myself as someone who enjoys artistic and aesthetic activities.

31. I see myself as someone who is sometimes shy, inhibited.

32. I see myself as someone who is considerate and kind to almost everyone.

33. I see myself as someone who is effective in his work.

34. I see myself as someone who stays calm in distressing situations.

35. I see myself as someone who prefers simple, routine work.

36. I see myself as

someone who is sociable, extroverted.

37. I see myself as someone who is sometimes rude to others.

38. I see myself as someone who makes plans and pursues them.

39. I see myself as someone who is easily anxious.

40. I see myself as someone who likes to think and play with ideas.

41. Je me vois comme quelqu'un qui est peu intéressé par tout ce qui est artistique.

41. I see myself as someone who has little interest in anything artistic.

43. I see myself as someone who is easily distracted.

44. I see myself as someone who has good knowledge of art, music or literature.

45. I see myself as someone looking for stories from others.

7. About everyday work: specify how often you feel the description of the following proposals. *

Une seule réponse possible par ligne.

	Jamais	Quelques fois dans l'année	Une fois par mois	Plusieurs fois par mois	Une fois par semaine	Plusieurs fois par semaine	Tous les jours
1. I feel nervously "emptied" by my work							
2. At the end of a work day, I sometimes feel completely exhausted.ail, il arrive que je me sente totalement épuisé(e).							
3. I feel tired when I get up in the morning and have to work another day.							
4. It is easy for me to understand how people feel.							
5. I have the impression of treating certain people as if they were impersonal "objects".							
6. Working with other people all day is a source of							

tension for me.							
7. I deal with people's problems very effectively.							
8. I feel totally washed out by my work.							
9. I believe that, through my professional activity, I have a positive influence on people's lives.							
10. I have the impression that since I started in the profession, I have become much harder and insensitive to others.							
11. I am afraid that my work will harden me.							
12. I feel full of energy.							
13. I feel frustrated in my work.							
14. I feel like I'm working too hard.							
15. I don't really care							

what happens to some people.

16. Working in direct contact with other people is an intense stress for me.	◯	◯	◯	◯	◯	◯	◯
17. With my colleagues, I can easily create a relaxed atmosphere.	◯	◯	◯	◯	◯	◯	◯
18. I feel plump, "revived" when I can follow people's cases closely.	◯	◯	◯	◯	◯	◯	◯
19. I consider myself to have accomplished many valuable acts in my work.	◯	◯	◯	◯	◯	◯	◯
20. I have the impression that I am “at the end of my rope”.	◯	◯	◯	◯	◯	◯	◯
21. In my work, I deal very calmly with emotional and emotional problems.	◯	◯	◯	◯	◯	◯	◯
22. I feel like most people make me	◯	◯	◯	◯	◯	◯	◯

responsible for some of their problems.

8. How many hours do you work on average per week? (Enter your weekly average in whole numbers) *

9. Mean number of night shifts per month :

Une seule réponse possible par ligne.

	Check the number of shifts
0	
1	
2	
3	
4	
5	
6	
7	
8	
9	
10	
11	
12	
>12	

10. Do you have the right to take a security rest after a night shift ? *

Une seule réponse possible.

- Yes
- No
- It depends on the working load

11. Since the beginning of your medical studies do you ever have suicidal thoughts ? *

Une seule réponse possible.

- Never
- Rarely
- Often
- Yes, with intention to act

12. Did you use ? *

Plusieurs réponses possibles.

- [ ] a therapist
- [ ] Performance enhancer
- [ ] antianxiety drug
- [ ] antidepressant
- [ ] sleeping pillls
- [ ] drugs
- [ ] nothing

13. What is your gender ? *

Une seule réponse possible.

- Male
- Female

14. What is your marital status ? *

Une seule réponse possible.

- Single
- Cohabitation
- "Pacsé(e)"
- Married
- Widow
- Divorced

15. Do you have kids ? *

Une seule réponse possible.

- Yes
- No

16. What is your profesionnal status ? *

Une seule réponse possible.

- resident
- fellow
- professor
- attending
- private practice

17. If you are a resident, in which year are you ?

Une seule réponse possible.

- 1st
- 2nd
- 3rd
- 4th
- 5th

18. Free comment

Ce contenu n'est ni rédigé, ni cautionné par Google.

Google Forms

Santé au travail de la communauté neurochirurgicale française

Chers collègues,

le métier de médecin est une activité éprouvante sur le plan physique et émotionnel. Son objectif est la préservation et l'amélioration de la santé des patients.
Seniors et internes de neurochirurgie, vous êtes engagés dans une spécialité où la notion de risque est inséparable de la pression qu'elle implique.
Le but de ce questionnaire est d'évaluer l'état de santé psychique de la communauté neurochirurgicale et ses déterminants, à l'aide d'outils académiques validés. L'objectif est de proposer des solutions pratiques et préventives aux difficultés rencontrées. Cette démarche s'inscrit dans le rapport du 5 novembre 2018 sur la recertification des médecins qui stipule que le médecin prend soin de sa santé et de sa qualité de vie.

Ce questionnaire est anonyme et les données recueillies sont sécurisées et utilisées aux seules fins de l'étude. Il a été déclaré à la CNIL (n° 2211947 v 0) et approuvé par le comité d'éthique du collège de neurochirurgie le 14/03/2019 (réf. IRB00011687). Le résultat de cette étude donnera lieu à une publication originale.

En vous remerciant pour votre collaboration à cette enquête (moins de 15 min) et restant à votre écoute pour tout renseignement complémentaire,

Clément Baumgarten – CHU Grenoble - Représentant des internes au Collège de neurochirurgie
Emmanuel Gay - CHU Grenoble - Président de la SFNC - PU-PH
Pierre-Hugues Roche – CHU Marseille - Président du Collège de neurochirurgie – PU-PH
Estelle Michinov – Université Rennes 2 - PU de psychologie
Géraldine Rouxel – Université Rennes 2 - MCU de psychologie

*Obligatoire

A propos du quotidien au travail: *

	Pas d'accord	D'accord, et je ne suis pas du tout perturbé(e)	D'accord, et je suis un peu perturbé(e)	D'accord, et je suis perturbé(e)	D'accord, et je suis très perturbé(e)
1. Je suis constamment pressé (e) par le temps à cause d'une forte charge de travail	○	○	○	○	○
2. Je suis fréquemment interrompu(e) et dérangé(e) dans mon travail	○	○	○	○	○
3. J'ai beaucoup de responsabilités à mon travail	○	○	○	○	○
4. Je suis souvent contraint(e) à faire des heures supplémentaires	○	○	○	○	○
5. Mon travail exige des efforts physiques	○	○	○	○	○
6. Au cours des dernières années, mon travail est devenu de plus en plus exigeant	○	○	○	○	○
7. On me traite injustement dans mon travail	○	○	○	○	○
8. Vu tous mes efforts, je reçois	○	○	○	○	○

le respect et l’estime que je mérite à mon travail

9. Je suis en train de vivre ou je m’attends à vivre un changement indésirable dans ma situation de travail	○	○	○	○	○
10. Mes perspectives de promotion sont faibles	○	○	○	○	○
11.Ma sécurité d'emploi est menacée	○	○	○	○	○
12. Au travail, il m'arrive fréquemment d'être pressé(e) par le temps	○	○	○	○	○
13. Je commence à penser à des problèmes au travail dès que je me lève le matin	○	○	○	○	○
14. Quand je rentre à la maison, j'arrive facilement à me décontracter et à oublier tout ce qui concerne mon travail	○	○	○	○	○
15.Mes proches disent que je me sacrifie trop pour mon travail	○	○	○	○	○

16. Le travail me trotte encore dans la tête quand je vais au lit ○ ○ ○ ○ ○

17. Quand je remets à plus tard quelque chose que je devrais faire le jour même, j'ai du mal à dormir le soir ○ ○ ○ ○ ○

A propos du quotidien au travail: *

	D'accord	Pas d'accord, et je ne suis pas du tout perturbé(e)	Pas d'accord, et je suis un peu perturbé(e)	Pas d'accord, et je suis perturbé(e)	Pas d'accord, et je suis très perturbé(e)
1. Je reçois le respect que je mérite de mes supérieurs	○	○	○	○	○
2. Je reçois le respect que je mérite de mes collègues	○	○	○	○	○
3. Au travail je bénéficie d'un soutien satisfaisant dans les situations difficiles	○	○	○	○	○
4. Ma sécurité d'emploi est menacé	○	○	○	○	○
5. Ma position professionnelle actuelle correspond bien à ma formation	○	○	○	○	○
6.Vu tous mes efforts, je reçois le respect et l'estime que je mérite à mon travail	○	○	○	○	○
7. Vu tous mes efforts, mes perspectives de promotion sont satisfaisantes	○	○	○	○	○

A propos du quotidien au travail: précisez la fréquence à laquelle vous ressentez la description des propositions suivantes. *

	Jamais	Presque jamais	Quelques fois	Régulièrement	Souvent	Très souvent	Toujours
1. Je voudrais encore faire ce travail, même si j'étais moins payé(e)	○	○	○	○	○	○	○
2. Quand je travaille, je ne pense à rien d'autre	○	○	○	○	○	○	○
3. Mon travail me procure des sentiments agréables	○	○	○	○	○	○	○
4. J'ai aussi envie de travailler durant mon temps libre	○	○	○	○	○	○	○
5. Je me laisse emporter par mon travail	○	○	○	○	○	○	○
6. Je fais mon travail avec beaucoup de plaisir	○	○	○	○	○	○	○
7. Je travaille parce que j'aime cela	○	○	○	○	○	○	○
8. Quand je	○	○	○	○	○	○	○

travaille, j’oublie tout le reste autour de moi

9. Je me sens heureux(se) pendant mon travail	○	○	○	○	○	○	○
10. Quand je travaille sur quelque chose, je le fais pour moi-même	○	○	○	○	○	○	○
11. Je suis totalement immergé(e) dans mon travail	○	○	○	○	○	○	○
12. Je me sens joyeux(se) quand je suis en train de travailler	○	○	○	○	○	○	○
13. Je tire ma motivation du travail en lui-même et non des récompenses que j’en reçois	○	○	○	○	○	○	○

A propos du quotidien au travail: précisez la fréquence à laquelle vous ressentez la description des propositions suivantes. *

	Jamais	Parfois	Souvent	Toujours
1. J'ai l'impression d'être pressé(e) et de courir contre la montre	○	○	○	○
2. Je reste à mon travail même après que mes collègues sont partis.	○	○	○	○
3. Il est important pour moi de travailler dur même quand je n'aime pas ce que je suis en train de faire	○	○	○	○
4. Je suis toujours occupé(e) et je travaille sur beaucoup de projets en même temps.	○	○	○	○
5. Il me semble que j'ai une compulsion interne qui me pousse à travailler dur, un sentiment que c'est quelque chose que je dois faire que je le veuille ou non.	○	○	○	○
6. Je passe plus	○	○	○	○

de temps à travailler qu'à passer du temps avec des ami(e)s, à avoir des hobbies ou à pratiquer des loisirs.

7. Je me sens obligé(e) de travailler dur même quand ce que je fais n'est pas agréable.	○	○	○	○
8. Je me retrouve à faire deux ou trois choses simultanément, comme, par exemple, à être en train de manger mon repas de midi et à rédiger une note en même temps que je suis au téléphone	○	○	○	○
9. Je me sens coupable quand j'ai congé au travail	○	○	○	○
10. Il m'est difficile de me détendre quand je ne suis pas en train de travailler	○	○	○	○

A propos des relations famille - travail *

	Tout à fait en désaccord	En désaccord	Plutôt en désaccord	Indécis(e)	Plutôt d'accord	D'accord	Tou[illegible] fa[illegible] d'ac[illegible]
1. Ma vie familiale empiète sur mes activités professionnelles	○	○	○	○	○	○	○
2. Ma vie familiale empiète sur ma vie professionnelle, m'empêchant d'arriver au travail à l'heure, d'accomplir mes tâches quotidiennes et de faire des heures supplémentaires	○	○	○	○	○	○	○
4. Mon travail m'empêche de faire tout ce que j'aimerais pouvoir faire chez moi	○	○	○	○	○	○	○
5. Mes partenaires et collègues de travail n'apprécient pas que je sois aussi souvent préoccupée par ma vie de famille	○	○	○	○	○	○	○
6. Il y a conflit entre mon travail et mes	○	○	○	○	○	○	○

responsabilités familiales							
7. Il me faut parfois m'absenter du travail pour pouvoir assumer mes responsabilités familiales	○	○	○	○	○	○	○
8. Mon travail m'empêche d'avoir une vie de famille harmonieuse	○	○	○	○	○	○	○
9. Je n'arrive pas à accomplir certaines tâches que mon travail implique à cause des sollicitations de ma famille/ mon conjoint	○	○	○	○	○	○	○
10. Il me faut souvent renoncer à des moments importants de la vie de famille à cause de mon travail	○	○	○	○	○	○	○

Je me considère comme une personne qui... *

	désapprouve fortement	désapprouve un peu	n'approuve ni ne désapprouve	approuve un peu	approuve fortement
1. Je me vois comme quelqu'un qui est bavard.	○	○	○	○	○
2. Je me vois comme quelqu'un qui a tendance à critiquer les autres.	○	○	○	○	○
3. Je me vois comme quelqu'un qui travaille consciencieusement.	○	○	○	○	○
4. Je me vois comme quelqu'un qui est déprimé, cafardeux.	○	○	○	○	○
5. Je me vois comme quelqu'un qui est créatif, plein d'idées originales.	○	○	○	○	○
6. Je me vois comme quelqu'un qui est réservé.	○	○	○	○	○
7. Je me vois comme quelqu'un qui est serviable et n'est pas égoïste avec les autres.	○	○	○	○	○
8. Je me vois comme quelqu'un qui peut être parfois négligent.	○	○	○	○	○
9. Je me vois comme quelqu'un qui est « relaxe », détendu, gère bien les stress.	○	○	○	○	○

10. Je me vois comme quelqu'un qui s'intéresse à de nombreux sujets.	◯	◯	◯	◯	◯
11. Je me vois comme quelqu'un qui est plein d'énergie.	◯	◯	◯	◯	◯
12. Je me vois comme quelqu'un qui commence facilement à se disputer avec les autres.	◯	◯	◯	◯	◯
13. Je me vois comme quelqu'un qui est fiable dans son travail.	◯	◯	◯	◯	◯
14. Je me vois comme quelqu'un qui peut être angoissé.	◯	◯	◯	◯	◯
15. Je me vois comme quelqu'un qui est ingénieux.	◯	◯	◯	◯	◯
16. Je me vois comme quelqu'un qui communique beaucoup d'enthousiasme.	◯	◯	◯	◯	◯
17. Je me vois comme quelqu'un qui est indulgent de nature.	◯	◯	◯	◯	◯
18. Je me vois comme quelqu'un qui a tendance à être désorganisé.	◯	◯	◯	◯	◯
19. Je me vois	◯	◯	◯	◯	◯

comme quelqu'un qui se tourmente beaucoup.

20. Je me vois comme quelqu'un qui a une grande imagination.	○	○	○	○	○
21. Je me vois comme quelqu'un qui a tendance à être silencieux.	○	○	○	○	○
22. Je me vois comme quelqu'un qui fait généralement confiance aux autres.	○	○	○	○	○
23. Je me vois comme quelqu'un qui a tendance à être paresseux.	○	○	○	○	○
24. Je me vois comme quelqu'un de tempéré, pas facilement troublé.	○	○	○	○	○
25. Je me vois comme quelqu'un qui est inventif.	○	○	○	○	○
26. Je me vois comme quelqu'un qui a une forte personnalité, s'exprime avec assurance.	○	○	○	○	○
27. Je me vois comme quelqu'un qui est parfois dédaigneux, méprisant.	○	○	○	○	○
28. Je me vois comme quelqu'un	○	○	○	○	○

qui persévère jusqu'à ce que sa tâche soit finie.

29. Je me vois comme quelqu'un qui peut être lunatique, d'humeur changeante.

30. Je me vois comme quelqu'un qui apprécie les activités artistiques et esthétiques.

31. Je me vois comme quelqu'un qui est quelquefois timide, inhibé.

32. Je me vois comme quelqu'un qui est prévenant et gentil avec presque tout le monde.

33. Je me vois comme quelqu'un qui est efficace dans son travail.

34. Je me vois comme quelqu'un qui reste calme dans les situations angoissantes.

35. Je me vois comme quelqu'un qui préfère un travail simple et routinier.

36. Je me vois comme quelqu'un qui est sociable, extraverti.

37. Je me vois

comme quelqu'un qui est parfois impoli avec les autres.

38. Je me vois comme quelqu'un qui fais des projets et les poursuit.	○	○	○	○	○
39. Je me vois comme quelqu'un qui est facilement anxieux.	○	○	○	○	○
40. Je me vois comme quelqu'un qui aime réfléchir et jouer avec des idées.	○	○	○	○	○
41. Je me vois comme quelqu'un qui est peu intéressé par tout ce qui est artistique.	○	○	○	○	○
42. Je me vois comme quelqu'un qui aime coopérer avec les autres.	○	○	○	○	○
43. Je me vois comme quelqu'un qui est facilement distrait.	○	○	○	○	○
44. Je me vois comme quelqu'un qui a de bonnes connaissances en art, en musique ou en littérature.	○	○	○	○	○
45. Je me vois comme quelqu'un qui cherche des histoires aux autres.	○	○	○	○	○

A propos du quotidien au travail: précisez la fréquence à laquelle vous ressentez la description des propositions suivantes. *

	Jamais	Quelques fois dans l'année	Une fois par mois	Plusieurs fois par mois	Une fois par semaine	Plusieurs fois par semaine	Tous les jours
1. Je me sens "vidé(e)" nerveusement par mon travail	○	○	○	○	○	○	○
2. A la fin d'une journée de travail, il arrive que je me sente totalement épuisé(e).	○	○	○	○	○	○	○
3. Je me sens fatigué(e) quand je me lève le matin et que je dois affronter une nouvelle journée de travail.	○	○	○	○	○	○	○
4. Il m'est facile de comprendre ce que ressentent les gens.	○	○	○	○	○	○	○
5. J'ai l'impression de traiter certaines personnes comme si elles étaient des "objets" impersonnels.	○	○	○	○	○	○	○
6. Travailler toute la journée avec d'autres	○	○	○	○	○	○	○

gens est pour moi source de tension.

7. Je m'occupe très efficacement des problèmes des gens. ○ ○ ○ ○ ○ ○ ○

8. Je me sens totalement lessivé par mon travail. ○ ○ ○ ○ ○ ○ ○

9. Je crois que, par mon activité professionnelle, j'exerce une influence positive sur la vie des gens. ○ ○ ○ ○ ○ ○ ○

10. J'ai l'impression que depuis mes début dans la profession, je suis devenu(e) nettement plus dur(e) et insensible envers autrui. ○ ○ ○ ○ ○ ○ ○

11. J'ai peur que mon travail ne m'endurcisse. ○ ○ ○ ○ ○ ○ ○

12. Je me sens plein(e) d' énergie. ○ ○ ○ ○ ○ ○ ○

13. Je me sens frustré(e) dans mon travail. ○ ○ ○ ○ ○ ○ ○

14. J'ai ○ ○ ○ ○ ○ ○ ○

l'impression de travailler trop dur.

15. Je ne me soucie pas vraiment de ce qui peut arriver à certaines personnes.	○	○	○	○	○	○	○
16. Travailler en contact direct avec d'autres gens représente pour moi un stress intense.	○	○	○	○	○	○	○
17. Avec mes collègues, je peux facilement créer une atmosphère détendue.	○	○	○	○	○	○	○
18. Je me sens regonflé(e), "ranimé(e)" quand je peux suivre de près le cas des gens.	○	○	○	○	○	○	○
19. J'estime avoir accompli de nombreux actes de valeur dans mon travail.	○	○	○	○	○	○	○
20. J'ai l'impression d'être « au bout du rouleau ».	○	○	○	○	○	○	○
21. Dans mon travail, je traite très calmement des problèmes	○	○	○	○	○	○	○

émotionnels et affectifs.

22. J'ai l'impression que la plupart des gens me rendent responsable de certains de leurs problèmes.

Combien d'heures travaillez-vous en moyenne par semaine ? (Inscrivez en chiffre entier votre moyenne hebdomadaire) *

Votre réponse

Nombre de gardes effectuées par mois (en moyenne):

	Cocher le nombre de garde
0	○
1	○
2	○
3	○
4	○
5	○
6	○
7	○
8	○
9	○
10	○
11	○
12	○
>12	○

Avez-vous droit au repos de garde ? *

- Oui
- Non
- Ca dépend de la charge de travail

Depuis le début de vos études, avez-vous eu des idées suicidaires ? *

- Jamais
- Raremement
- Souvent
- Oui, avec intention de passage à l'acte

Avez-vous eu recours à: *

- [ ] un thérapeute (psychiatre, psychologue...)
- [ ] des produits dopants
- [ ] des anxiolytiques
- [ ] des antidépresseurs
- [ ] des somnifères
- [ ] des stupéfiants (cannabis, cocaïne...)
- [ ] rien

Quel est votre sexe ? *

- Homme
- Femme

Quel est votre statut marital ? *

- Célibataire
- En concubinage
- Pacsé(e)
- Marié(e)
- Veuf
- Divorcé

Avez-vous des enfants ? *

- Oui
- Non

Quel est votre statut professionnel ? *

- Interne
- Assistant/Chef de clinique
- PH
- MCU-PH
- PU-PH
- Praticien libéral

Si vous êtes interne, en quelle année êtes-vous ?

- 1ère
- 2ème
- 3ème
- 4ème
- 5ème

Commentaire libre: réflexion personnelle, partage d'expérience, suggestion...

Votre réponse

Envoyer

N'envoyez jamais de mots de passe via Google Forms.

Ce contenu n'est ni rédigé, ni cautionné par Google. Signaler un cas d'utilisation abusive - Conditions d'utilisation - Règles de confidentialité

Google Forms
