# Supplementary material for: Personal and psychosocial factors of burnout: A survey within the French neurosurgical community
Source: PLoS One. 2020 May 29;15(5):e0233137. doi: 10.1371/journal.pone.0233137 (PMC7259549; doi:10.1371/journal.pone.0233137)
Supplement: S1 Table — Alphas on the diagonal: a after elimination of item 20; b: after elimination of items 35 and 41. (DOCX) [file pone.0233137.s002.docx]

**S1 Table. Cronbach Alphas**

|  | 1. | 2. | 3. | 4. | 5. | 6. | 7. | 8. | 9. | 10. | 11. | 12. | 13. | 14. | 15. | 16. | 17. | 18. | 19. |
| --- | --- | --- | --- | --- | --- | --- | --- | --- | --- | --- | --- | --- | --- | --- | --- | --- | --- | --- | --- |
| 1. Burnout Emotional Exhaustion | .898 |  |  |  |  |  |  |  |  |  |  |  |  |  |  |  |  |  |  |
| 2. Burnout Personal Achievement |  | .843 |  |  |  |  |  |  |  |  |  |  |  |  |  |  |  |  |  |
| 3. Burnout Depersonalization |  |  | .711 |  |  |  |  |  |  |  |  |  |  |  |  |  |  |  |  |
| 4. Effort |  |  |  | .786 |  |  |  |  |  |  |  |  |  |  |  |  |  |  |  |
| 5. Reward |  |  |  |  | .781 |  |  |  |  |  |  |  |  |  |  |  |  |  |  |
| 6. Overinvestment |  |  |  |  |  | .81^a^ |  |  |  |  |  |  |  |  |  |  |  |  |  |
| 7. Effort/Reward ratio |  |  |  |  |  |  |  |  |  |  |  |  |  |  |  |  |  |  |  |
| 8. Flow Absorption |  |  |  |  |  |  |  | .765 |  |  |  |  |  |  |  |  |  |  |  |
| 9. Flow Pleasure |  |  |  |  |  |  |  |  | .91 |  |  |  |  |  |  |  |  |  |  |
| 10. Flow Intrinsic Motivation |  |  |  |  |  |  |  |  |  | .758 |  |  |  |  |  |  |  |  |  |
| 11. Excessive work |  |  |  |  |  |  |  |  |  |  | .744 |  |  |  |  |  |  |  |  |
| 12. Compulsive work |  |  |  |  |  |  |  |  |  |  |  | .787 |  |  |  |  |  |  |  |
| 13. Family Work Conflict |  |  |  |  |  |  |  |  |  |  |  |  | .853 |  |  |  |  |  |  |
| 14. Work Family Conflict |  |  |  |  |  |  |  |  |  |  |  |  |  | .774 |  |  |  |  |  |
| 15. Extraversion |  |  |  |  |  |  |  |  |  |  |  |  |  |  | .824 |  |  |  |  |
| 16. Agreeableness |  |  |  |  |  |  |  |  |  |  |  |  |  |  |  | .771 |  |  |  |
| 17. Conscientiousness |  |  |  |  |  |  |  |  |  |  |  |  |  |  |  |  | .807 |  |  |
| 18. Neuroticism |  |  |  |  |  |  |  |  |  |  |  |  |  |  |  |  |  | .794 |  |
| 19. Openness |  |  |  |  |  |  |  |  |  |  |  |  |  |  |  |  |  |  | .81^b^ |

Alphas on the diagonal: ^a^ after elimination of item 20; ^b^: after elimination of items 35 and 41
