# Supplementary material for: Personal and psychosocial factors of burnout: A survey within the French neurosurgical community
Source: PLoS One. 2020 May 29;15(5):e0233137. doi: 10.1371/journal.pone.0233137 (PMC7259549; doi:10.1371/journal.pone.0233137)
Supplement: S2 Table — (DOCX) [file pone.0233137.s003.docx]

**S2 Table.** **Variables entered during the stepwise multiple regression**

| Questionnaire | Item |
| --- | --- |
| Siegrist Effort/Reward scale | Effort/Reward Ratio |
|  | Overinvestment |
| DUWAS scale | Excessive work |
|  | Compulsive work |
| Flow | Flow Absorption |
|  | Flow Pleasure |
|  | Flow Intrinsic Motivation |
| Work-Family conflict scale | Family Work Conflict |
|  | Work Family Conflict |
| Big Five inventory | Extraversion |
|  | Agreeableness |
|  | Conscientiousness |
|  | Neuroticism |
|  | Openness |
| Mean declared worked hours per week |  |
| Mean declared night shifts per month |  |
| Gender |  |
| Hierarchical status |  |
| Marital status |  |
| Having Child |  |
