# Supplementary figures and images for: Personal and psychosocial factors of burnout: A survey within the French neurosurgical community
Source: PLoS One. 2020 May 29;15(5):e0233137. doi: 10.1371/journal.pone.0233137 (PMC7259549; doi:10.1371/journal.pone.0233137)

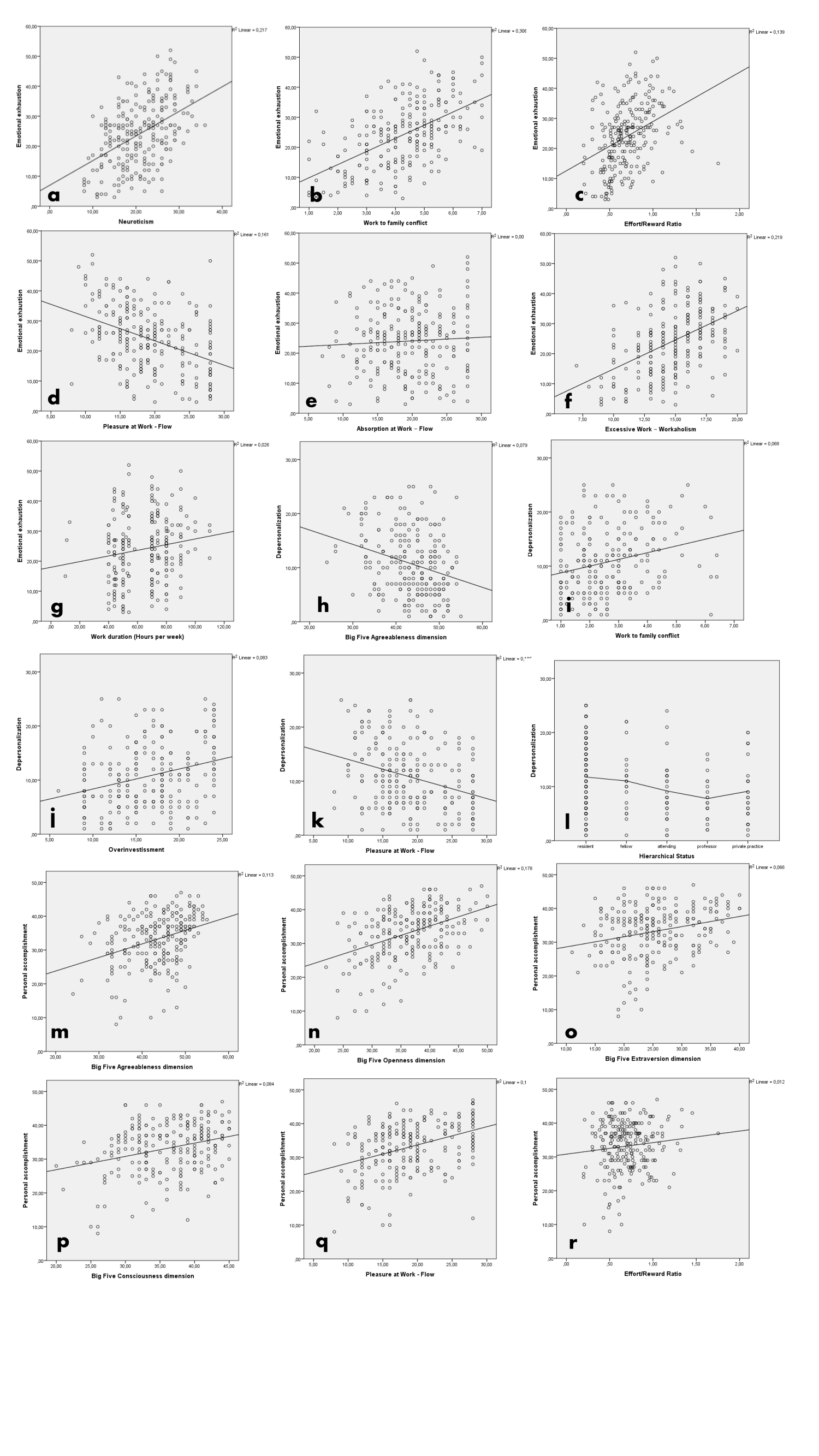

Supplement: S1 Fig — Factors associated with emotional exhaustion: a, b, c, d, e, f, g. a: linear regression between emotional exhaustion and pleasure at work; b: linear regression between emotional exhaustion and neuroticism; b: linear regression between emotional exhaustion and work to family conflict; c: linear regression between emotional exhaustion and effort/reward ratio; d: linear regression between emotional exhaustion and pleasure at work; e: linear regression between emotional exhaustion and absorption at work; f: linear regression between emotional exhaustion and excessive work; g: linear regression between emotional exhaustion and work duration. Factors associated with depersonalization: h, i, j, k, l. h: linear regression between depersonalization and agreeableness; i: linear regression between depersonalization and work to family conflict; j: linear regression between depersonalization and overinvestment; k: linear regression between depersonalization and pleasure at work; l: linear regression between depersonalization and hierarchical status. Factors associated with personal accomplishment: m, n, o, p, q, r. m: linear regression between personal accomplishment and agreeableness; n: linear regression between personal accomplishment and openness; o: linear regression between personal accomplishment and extraversion; p: linear regression between personal accomplishment and consciousness; q: linear regression between personal accomplishment and pleasure at work; r: linear regression between personal accomplishment and effort/reward ratio. (TIFF) [file pone.0233137.s004.tiff]
