# Supplementary material for: Personal and psychosocial factors of burnout: A survey within the French neurosurgical community
Source: PLoS One. 2020 May 29;15(5):e0233137. doi: 10.1371/journal.pone.0233137 (PMC7259549; doi:10.1371/journal.pone.0233137)
Supplement: Attachment — Submitted filename: Response to Reviewers.docx [file pone.0233137.s006.docx]

Editors

Plos ONE

Ref : PONE-D-20-00752R1

Personal and Psychosocial factors of Burnout: A Survey within the French Neurosurgical Community

To Sergio A. Useche, Academic Editor

Dear Sir,

Please find enclosed a revised manuscript entitled “Personal and Psychosocial factors of Burnout: A Survey within the French Neurosurgical Community” by Clément Baumgarten, Estelle Michinov, Géraldine Rouxel, Vincent Bonneterre, Emmanuel Gay and Pierre-Hugues Roche. This manuscript is submitted for consideration after revisions for publication in the PLOS ONE journal. We have addressed and clarified all the concerns that were raised by the academic editor and the reviewers. We believe that this revised version results in a much stronger paper for your journal. All the authors have reviewed and approved this revised version of the manuscript.

In addition to the revised version of our manuscript (in Word format), please find enclosed the following documents:

- A point-by-point list of all changes made in response to suggestions of the reviewers.
- A version of the manuscript in “track changes” format, with changes highlighted (in Word format).

In the hope that you find that this paper falls within the scope of PLOS ONE, and that it will be of interest to your readers, I look forward to hearing from you soon. Please do not hesitate to contact me if you have any questions.

Yours faithfully,

Clément Baumgarten

**Response to reviewers**

Ref: PONE-D-20-00752R1

Personal and Psychosocial factors of Burnout: A Survey within the French Neurosurgical Community

I. **INTRODUCTION**

The authors would like to thank the reviewers for their relevant remarks and suggestions for improving the paper. In this document, we provide a point-by-point response to each of the issues raised. Each issue is written in bold text, the corresponding response is in normal text, and we provide in italics the text added/modified in the paper to address the issue.

Each modification in the text of the manuscript was highlighted in red to be more easily visible for the reviewers.

Here is a point-by-point list of all changes made in response to the suggestions of the reviewers:

II. **REVIEWER 1**

**The research is relevant. The burnout syndrome is a disease that affects various professional categories and grow the number of research on this subject. In the Abstract the authors reported a high prevalence of burnout in French Neurosurgeons. However, in the final conclusion the authors did not make any reference to the burnout levels in this category of professionals. Also i did not realise the scale wich indicates the burnout levels are high or low.**

**Please, check it.**

This is indeed a relevant point in the move of our paper that was missing. We compared our results with a meta-analysis of French physicians and American surgeons. Our conclusion of high prevalence of burnout was drawn on this comparison because such scale does not exist. Burnout definition and high, medium, or low level of each subscales (i.e. emotional exhaustion, depersonalization and personal accomplishment) were based on [1].

1.	Maslach C, Jackson SE. The measurement of experienced burnout. J Organ Behav. 1981;2: 99–113. doi:10.1002/job.4030020205

*Added text line 196: which is higher than the 29 % rate in comparison with the most comprehensive and complete meta-analysis of French physicians[17] following the same burnout definition. As for surgeons, Campbell et al[18] reported a 32% rate in a population of American surgeons.*

*Added text line 300: Burnout prevalence is high in comparison with physicians and even among surgeons.*
